# Influence of artificial intelligence on ophthalmologists’ judgments in glaucoma

**DOI:** 10.1371/journal.pone.0321368

**Published:** 2025-04-16

**Authors:** Kenji Kashiwagi, Masahiro Toyoura, Xiaoyang Mao, Kazuhide Kawase, Masaki Tanito, Toru Nakazawa, Atsuya Miki, Kazuhiko Mori, Takeshi Yoshitomi

**Affiliations:** 1 Department of Ophthalmology, University of Yamanashi Faculty of Medicine, Chuo, Japan; 2 Department of Computer Science and Engineering, University of Yamanashi, Kofu, Japan,; 3 Yasuma Eye Clinic, Nagoya, Japan,; 4 Department of Ophthalmology Protective Care for Sensory Disorders, Nagoya University Graduate School of Medicine, Nagoya, Japan; 5 Department of Ophthalmology, Shimane University Faculty of Medicine, Izumo, Japan; 6 Department of Ophthalmology, Tohoku University Graduate School of Medicine, Sendai, Japan; 7 Department of Myopia Control Research, Aichi Medical University, Nagoya, Japan; 8 Baptist Eye Institute, Kyoto, Japan,; 9 Department of Ophthalmology, Kyoto Prefectural University of Medicine, Kyoto, Japan; 10 Department of Orthoptics, Fukuoka International University of Health and Welfare, Fukuoka, Japan; Alexandria University Faculty of Medicine, EGYPT

## Abstract

**Purpose:**

To examine the influence of artificial intelligence (AI) on physicians’ judgments regarding the presence and severity of glaucoma on fundus photographs in an online simulation system.

**Methods:**

Forty-five trainee and expert ophthalmologists independently evaluated 120 fundus photographs, including 30 photographs each from patients with no glaucoma, mild glaucoma, moderate glaucoma, and severe glaucoma. A second trial was conducted at least one week after the initial trial in which photograph presentation order was randomized. During the second trial, 30% of the glaucoma judgments made by the AI system were intentionally incorrect. The evaluators were asked about their thoughts on AI in ophthalmology via a 3-item questionnaire.

**Results:**

The percentage of correct responses for all images significantly improved (P < 0.001) from 48.4 ± 24.8% in the initial trial to 59.6 ± 20.3% in the second trial. The improvement in the correct response rate was significantly greater for trainees (14.2 ± 19.0%) than for experts (8.6 ± 11.4%) (P = 0.04). The correct response rate was 63.9 ± 20.6% when the AI response was correct, significantly greater than the 47.9 ± 26.6% when the AI response was incorrect (P < 0.0001). For trainees, the correct response rate was significantly greater when the AI’s response was correct than when it was incorrect. However, for experts, the effect was less pronounced. The decision time was significantly longer when the AI response was incorrect than when it was correct (P = 0.003).

**Conclusion:**

In fundus photography-based glaucoma detection, the results of AI systems can influence physicians’ judgments, particularly those of physicians with less experience.

## Introduction

In recent years, artificial intelligence (AI)-based imaging algorithms have demonstrated high accuracy, equivalent to or even superior to that of ophthalmologists, in the diagnosis of many ophthalmic diseases [1–7]. The development of better-performing AI algorithms is expected to improve the level of medical care, but several challenges remain in the use of AI in clinical practice. Neither AI systems nor physicians have 100% diagnostic accuracy, and therefore some misdiagnoses will still be made. For image-based diagnoses made by a physician, diagnostic guidelines can be referenced so that other physicians and healthcare workers can confirm the diagnosis. However, when AI is involved, the basis for the diagnosis may not be clear; this lack of transparency has become a factor in physicians’ distrust of AI [8]. Neural networks and deep learning-based AI algorithms lack clarity and transparency, but to ensure physician satisfaction, it is crucial that these algorithms be made accountable for the results of their analyses. In many diagnostic imaging procedures, the accuracy of the judgments of the AI system is similar to or even better than that of a human physician; therefore, a physician with high diagnostic skills is still needed to verify whether the AI has given an incorrect response. Furthermore, since AI is often reported to have high diagnostic accuracy, it is possible that the diagnostic results of AI may influence physician judgment, resulting in a bias in the final diagnosis.

Early detection of glaucoma is extremely important, as subjective symptoms are often rare, and the damage from the disease is irreversible. Although fundus photographs are useful for diagnosing glaucoma, Soh et al. reported that the incidence of undetected glaucoma has not improved in recent years [9]. This may be because accurate determination of glaucoma largely depends on the diagnostic ability of the physician and because the lack of a diagnostic environment hinders proper diagnosis by ophthalmologists. Many recent studies have reported the high accuracy of AI in diagnosing glaucoma via fundus photographs; therefore, AI is expected to be useful in the management of glaucoma patients. However, its increasing use may lead to two major problems. First, if an AI system misdiagnoses a patient, the ophthalmologist may overlook the patient, which can lead to delayed diagnosis and disease progression. Second, the ophthalmologists’ ability to diagnose glaucoma may decrease if they begin relying more heavily on the AI system in their diagnosis. This reduced diagnostic capacity may have a negative impact on providing appropriate glaucoma management.

A previous study revealed that the diagnostic accuracy of general practitioners was influenced by a fictitious AI diagnostic support system in the diagnosis of skin lesions. When the AI provided the correct diagnosis, the physician’s diagnostic ability improved. However, when the AI provided the wrong information, only a small percentage of the physicians were able to disagree with the AI [10]. Since the impact of AI on ophthalmologists’ judgment in the diagnosis of glaucoma has not been determined, we used an online simulation system referred to as the Fictitious AI-based Diagnosis System to examine the influence of AI on ophthalmologists’ ability to detect and assess the severity of glaucoma on fundus photographs.

## Subjects and methods

This study was approved by the Ethics Committee of the University of Yamanashi School of Medicine (Approval Code 2635). The study was performed in accordance with the Declaration of Helsinki. All the images used in this study have been anonymized, and the ethics committee has waived the requirement for informed consent. Investigators accessed the data for research purposes on 09/03/2023.

### Fundus photograph subjects

Consecutive patients aged 40–70 years who attended the Department of Ophthalmology, University of Yamanashi Hospital, between January and June 2021 were considered for the study. The inclusion criteria were as follows: phakia coupled with best corrected visual acuity of 18/20 or better, refractive error within +3.0 D to −6.0 D, and clear fundus color photographs, while control eyes had to present with no ocular diseases other than mild cataracts. The exclusion criteria for the fundus images were as follows: unclear focus, smudged peripheral areas, exclusion of the papillae and the posterior pole, inclusion of eyelashes and other artifacts, and poor image quality as judged by the main author (KK).

### Image acquisition

24-2 testing was performed with a Humphrey visual field analyzer (HFA) (Carl-Zeiss Medics, Tokyo, Japan) during the entry period, employing visual field tests with solid vision failure, false negative, and false positive rates of less than 20%. Fundus color photography was performed with a fundus camera (CR-2 AF, CANON, Tokyo, Japan); information such as personal identifiers, angle of view information, and date and time of acquisition was removed from the photographs prior to analysis.

The images were divided into four severity groups, namely, nonglaucomatous, mildly glaucomatous, moderately glaucomatous and severely glaucomatous eyes, with 30 images assigned to each group. Glaucoma severity was defined as mild if the mean deviation according to the HFA 24-2 test was better than −6.0 dB, moderate if it was between −6.0 dB and −12.0 dB, and severe if it was worse than −12.0 dB.

### Image evaluators

A total of 49 ophthalmologists who understood the purpose of the study and agreed to cooperate in the research initially participated as evaluators: 26 had at least five years of clinical ophthalmology experience and had achieved an ophthalmology specialization (ophthalmology experts), and 23 were in the process of obtaining an ophthalmology specialization (within two years, ophthalmology trainees). Four evaluators were excluded from the study because of protocol violations; therefore, a total of 45 evaluators were included in the final analysis.

### Evaluation

#### Initial assessment.

**Fig 1:**
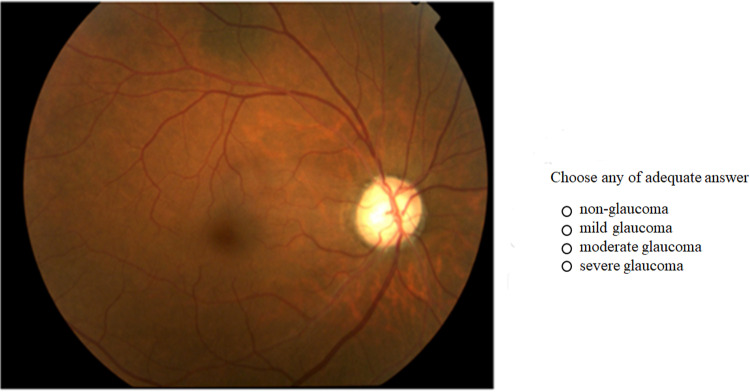
Representative screenshot of the internet-based judgment system for the first trials.

Both the initial and second trials were performed with an online decision system developed for this study by the Department of Computer Science and Engineering, Faculty of Engineering, University of Yamanashi. A representative example of the presentation screen and fundus images is shown in Fig 1. Thirty images from each of the four previously described severity groups were presented in a random order on a computer monitor; the evaluator was given 30 seconds to assess each fundus photo to determine whether glaucoma was present and, if so, whether the glaucoma was mild, moderate, or severe. The evaluator was instructed to assess the images with breaks to allow sufficient time to concentrate on the assessment.

#### Reassessment.

**Fig 2:**
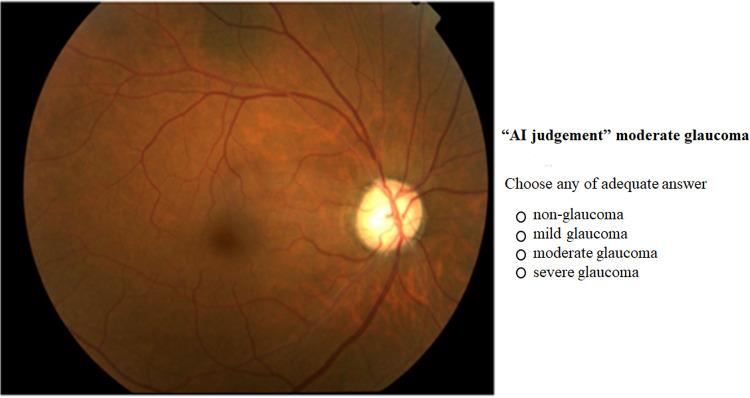
Representative screenshot of the internet-based judgment system used for the second trials.

A second trial involving the same instructions was performed at least one week after the initial trial, but the order of the presentation of the images was randomly rearranged. Supplementary information in the form of the AI judgment, termed the mimic AI system, was presented on the monitor, in contrast to the initial image (Fig 2).

### AI judgment results in the online diagnostic system

Among the AI judgment results presented during the second trial, 30% were intentionally incorrect. The results of the mimic AI system shown onscreen are summarized in Table 1.

**Table 1:** Criteria for presenting incorrect judgment outcomes.

Correct answer	Actual presentation
Non-glaucoma	30% presented as mild glaucoma
Mild glaucoma	15% presented as non-glaucoma 15% presented as moderate glaucoma
Moderate glaucoma	15% presented as mild glaucoma 15% presented as severe glaucoma
Severe glaucoma	30% presented as moderate glaucoma

### Questionnaire survey of evaluators

After the second trial, a questionnaire consisting of 3 items answered via a 4-point Likert scale (none, little, mild, strong) was administered to the evaluators. The questionnaire asked the following questions: How much influence did the AI response have on their second judgment? Do they feel threatened by the use of AI in ophthalmology? Do they have expectations for the use of AI in ophthalmology?

### Statistical analysis

A paired t test (or Wilcoxon rank-sum test for unpaired data) was used to analyze the judgments and response times between the two groups. The Steel–Dwass test (multiple analyses of nonparametric comparisons) was used to assess significant differences among the nonglaucoma, mild impairment, moderate impairment, and severe impairment groups. Repeated-measures analysis of variance (ANOVA) was performed via JMP17 software (JMP Statistical Discovery LLC, USA), and the significance level for all tests was set at P < 0.05.

## Results

### Rates of correct responses in the initial and second trials

**Fig 3:**
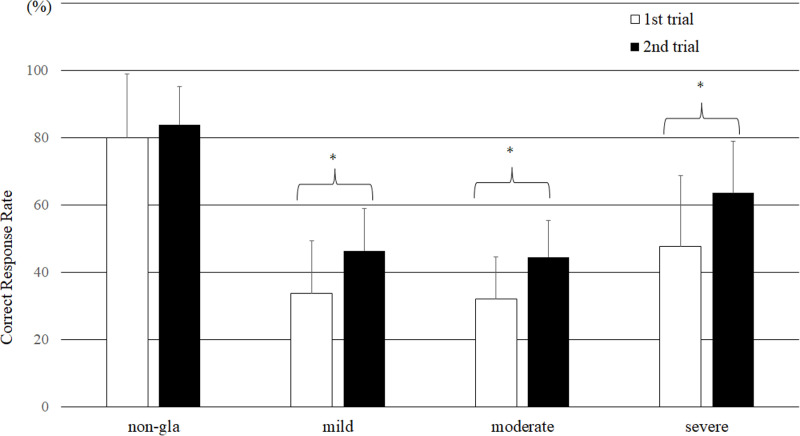
Comparison of the correct response rates between the first and second trials. (Paired *t* test, *P<0.0001 between two trials) (bar = SD).

Fig 3 shows the rate of correct responses for the first and second trials. For all the evaluators, the correct response rate (CRR) for all the images was 48.4 ± 24.8% in the first trial, and significant improvements were seen in the second trial, with a CRR of 59.6 ± 20.3% (P < 0.001). In both trials, the CRR was highest in diagnosing nonglaucoma, followed by severe glaucoma. The CRR in the second trial was significantly greater than that in the first trial in all patient groups except for the nonglaucoma group (P < 0.001).

### Comparison of the correct response rates between experts and trainees

**Fig 4:**
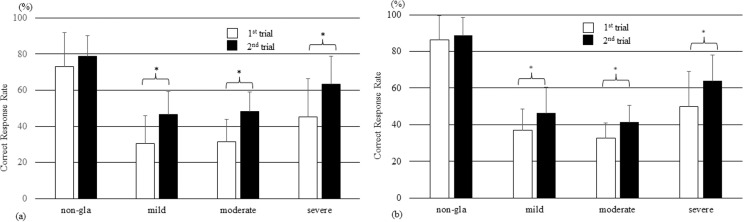
Comparison of expert and trainee correct response rates in the first and second trials. The rates of correct responses in the first and second trials were compared between experts (a) and residents (b). (Paired *t* test, *P<0.001 between two trials) (bar = SD).

Both experts and trainees showed significant improvements in the CRR between the first and second trials in all severity groups except the nonglaucoma group (Figs 4a and b).

**Fig 5:**
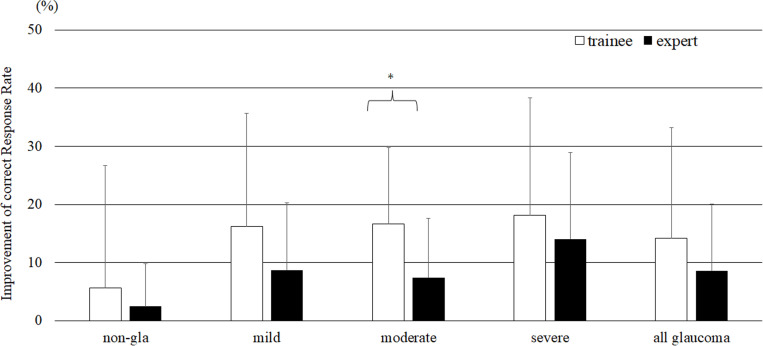
Comparison of accuracy improvements between trainees and experts (Steel–Dwass test, *P = 0.03) (bar = SD).

The improvement in the CRR between the first and second trials was significantly greater for trainees (14.2 ± 19.0%) than for experts (8.6% ± 11.4%, P = 0.04), and the trainees tended to show greater performance for all severity groups. A significant improvement (P = 0.03) was observed for the moderate glaucoma severity group (Fig 5).

### Impact of the supplementary presentation of AI results on the CRRs of the evaluators

**Fig 6:**
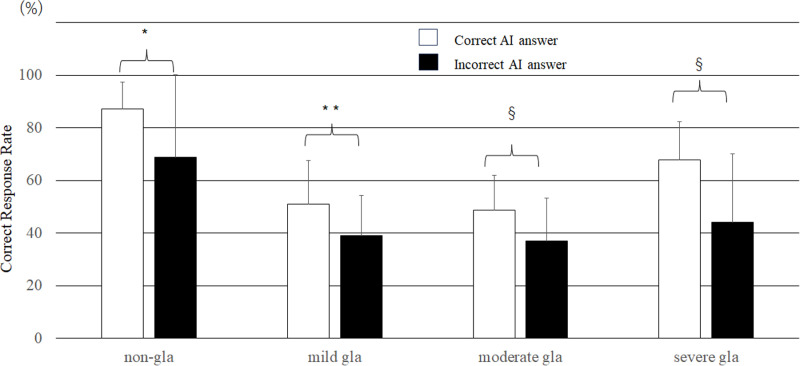
Effects of the mimic AI system results on the correct response rate. (Steel–Dwass test, *P = 0.03, **P = 0.003, §P < 0.0001) (bar = SD).

For all the evaluators, the CRR for all severity groups was 63.9 ± 20.6% when the mimic AI system gave a correct answer, significantly greater than the CRR of 47.9 ± 26.6% when the mimic AI system gave an incorrect answer (P < 0.0001). As shown in Fig 6, the CRRs for all groups were significantly greater when the AI mimic system gave a correct response than when it gave an incorrect response. For trainees, the CRR for all severity groups was 66.5 ± 18.5% when the AI mimic system gave a correct answer, significantly greater than the 41.5 ± 18.5% CRR when the AI mimic system gave an incorrect answer (P < 0.0001). Across all groups, the CRR was significantly greater when the AI mimic system indicated a correct answer than when it indicated an incorrect answer. The experts tended to have higher CRRs when the AI mimic system indicated a correct answer than when it indicated an incorrect answer for all severity groups, but the differences were not statistically significant (Table 2). The rate of improvement for images with correct responses was 24.9 ± 26.7% for trainees and 9.7 ± 21.7% for experts, and this difference was significant (P < 0.0001). The improvement rate was significantly better for trainees than for experts in all severity groups, and the most significant difference was found in the severe glaucoma group (Fig 6).

**Table 2:** Comparison of the effects of the AI judgment results on the CRR between experts and trainees.

Trainee	correct AI answer	incorrect AI answer	P value	Expert	correct AI answer	incorrect AI answer	P value
All	66.5 ± 18.5	41.5 ± 18.5	<0.0001	All	62.3 ± 22.4	52.7 ± 27.1	0.017
Non-glaucoma	84.6 ± 8.9	51.5 ± 34.8	0.003	Non-glaucoma	89.7 ± 10.4	81.0 ± 22.6	0.4
Mild-glaucoma	55.6 ± 17.8	37.3 ± 13.5	0.001	Mild-glaucoma	49.0 ± 16.3	42.1 ± 17.4	0.312
Moderate-glaucoma	55.3 ± 12.1	39.9 ± 20.0	0.001	Moderate-glaucoma	43.8 ± 11.9	36.2 ± 16.1	0.18
Severe-glaucoma	70.5 ± 16.1	37.7 ±± 25.7	<0.0001	Severe-glaucoma	66.8 ± 14.0	51.5 ±± 26.7	0.075

### Comparison of CRRs when the AI mimic system showed an incorrect result

**Fig 7:**
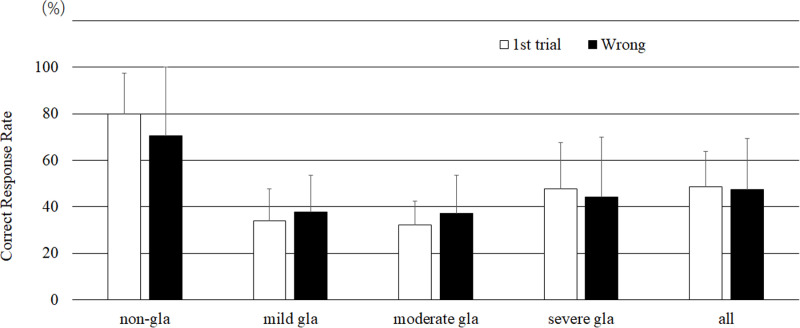
Correct response rate comparison between the first and second trials with incorrect mimic AI system results (Steel–Dwass test) (bar = SD).

During the second trial, the CRRs obtain when incorrect AI mimic system responses were shown were compared to the CRRs obtained during the first trial. No significant difference was found between the first and second CRRs for all evaluators (Fig 7).

### Analysis of response times

**Fig 8:**
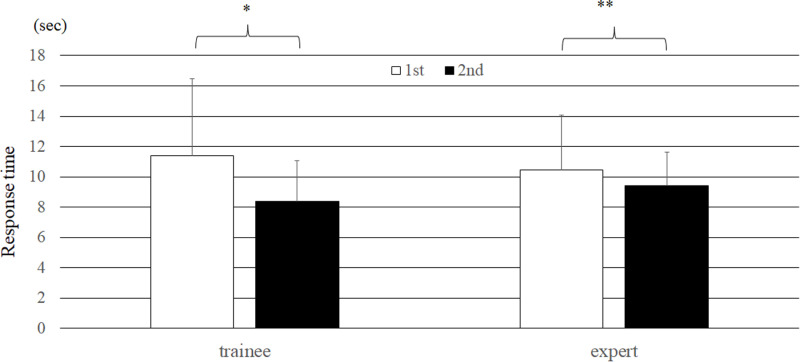
Comparison of response times between experts and trainees (bar = SD).

Overall, the evaluators had a significantly shorter response time in the second trial (9.0 ± 2.5 sec) than in the first trial (10.8 ± 4.3 sec, P = 0.0005). For trainees, the response time in the second trial (11.4 ± 5.1 sec) was significantly shorter than that in the first trial (8.4 ± 2.7 sec, P < 0.0001). Additionally, the response times among the experts for the first and second trials were 10.4 ± 3.7 seconds and 9.4 ± 2.2 seconds, respectively (P = 0.009). The reduction in response time for the second trial relative to the first trial was 2.4 ± 3.9 sec for trainees and 1.0 ± 2.7 sec for experts; this difference was statistically significant (P < 0.0001) (Fig 8).

### Relationship between the number of correct responses and response time

The response times of all the evaluators for correct and incorrect responses were also compared. In the first trial, the response time for correct responses was 10.0 ± 3.7 seconds, and the response time for incorrect responses was 11.7 ± 4.7 seconds; however, this difference was not significant (P=0.063). In the second trial, the difference in response times for correct responses (8.2 ± 2.0 seconds) and incorrect responses (9.7 ± 2.7 seconds) was significant (P = 0.003).

### Comparison of response times between correct and incorrect ai answers in the second trial

**Fig 9:**
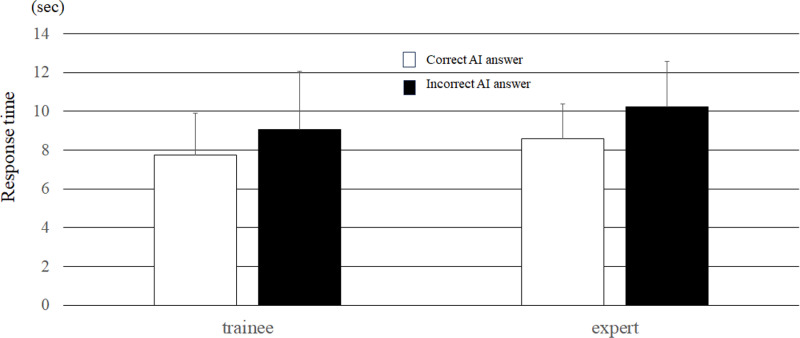
Comparison of response times between correct and incorrect AI responses. (Paired *t* test, *P < 0.001, **P = 0.009 between two trials) (bar = SD).

The response times when the AI mimic system presented a correct response and when the AI mimic system presented an incorrect response were compared. For all the evaluators, the response time was 9.31 ± 2.9 s when the AI mimic response was incorrect and 8.5 ± 2.3 s when the AI mimic system response was correct; this difference was statistically significant (P = 0.001). When comparing experts and trainees, the response times were longer for both groups when the AI mimic system indicated an incorrect response; this tendency was more pronounced for experts. The difference in response times among the experts was 1.64 seconds, which was slightly longer than the 1.32 seconds observed among the trainees (Fig 9).

### Results of the questionnaire administered after the second trial (Table 3)

The most common choice of both the trainees and experts was that the response of the mimic AI system had a mild influence on their decisions. The trainees tended to consider themselves more influenced than the experts, but the difference was not significant. A greater proportion of trainees felt threatened by the future use of AI in ophthalmic practice. On the other hand, the experts were more likely to have higher expectations of AI. Overall, the trainees were more concerned about the use of AI in medicine than the experts were.

**Table 3:** Comparison of questionnaire results between experts and trainees.

		None (%)	Little (%)	Mild (%)	Strong (%)
Effect on judgement	expert	0	16.7	55.6	27.8
	trainee	3.9	26.9	53.9	15.4
Thread to AI	expert	19.2	61.8	15.4	3.8
	trainee	0	55.6	33.3	11.1
Hope to AI	expert	7.7	0	26.9	65.4
	trainee	33.6	11.1	33.3	22.2

## Discussion

In this study, the impact of AI diagnosis on ophthalmologists’ judgments—which, to date, had not yet been explored—was investigated via a simulation system. The results showed that the AI system’s judgments influenced ophthalmologists’ diagnoses and that this influence was particularly strong for ophthalmologists with fewer years of experience. The accuracy of ophthalmologists’ judgments regarding the presence of glaucoma was relatively high, but the accuracy of judging the severity of glaucomatous optic neuropathy was not sufficiently high. Furthermore, ophthalmologists’ acceptance of the use of AI in glaucoma care was relatively positive.

In recent years, the diagnostic capabilities of AI have improved rapidly, with many reports indicating that these capabilities are equal to or even better than those of a physician [4,6,11–14]. Because of its promising results, AI has begun to be used in clinical settings; therefore, it is essential that physicians are also aware of its negative impact on their diagnostic ability.

There have been several reports in other fields on the impact of AI on physicians’ diagnostic abilities [15,16]. Nagendram et al. reported that explainable AI information influences physicians prescriptions [17], whereas Jacob et al. reported that incorrect AI judgments may adversely impact clinicians’ treatment selections [18]. Micocci et al. conducted a study using a fictitious AI diagnostic support system and reported that the accuracy of skin lesion diagnoses by general practitioners improved when the AI gave a correct answer; however, they did not disagree with the AI when presented with an incorrect answer [10]. In the field of ophthalmology, there is little research on how AI-based diagnostic outcomes affect ophthalmologists. Previous studies have demonstrated that AI systems can assess glaucoma with a high degree of accuracy [2,5,6,19]. In this study, we investigated whether the AI system influenced physicians’ clinical judgments. Evaluators were informed that the system is capable of making highly accurate assessments regarding both the presence and severity of glaucoma, consistent with findings from previous studies5. While the AI system used in this study was trained on expert evaluations by ophthalmologists, we aimed to determine whether providing this information would impact the evaluators’ decision-making process.

In this study, we showed not only that ophthalmology trainees with insufficient diagnostic abilities were more likely to rely on the judgments provided by AI but also that experts could also be influenced to the decisions of such systems. The response time for trainees was shorter than that for experts regardless of whether the AI judgment was correct or incorrect, which further suggests that trainees are more likely to rely on AI judgment. Stewart et al. reported that physicians may depend entirely on AI systems and not make any personal judgments [20]. Therefore, ophthalmologists, especially those with less experience, must continue training until they become experts in handling AI systems properly.

The introduction of AI into glaucoma practice has demonstrated several advantages [5,6,14,19]. For example, AI may improve diagnostic ability and prevent misdiagnosis. In the present study, the evaluators showed relatively high performance in distinguishing between fundus images with and without glaucoma, but their ability to evaluate the severity of glaucoma was relatively poor. In future AI studies, the assessment of disease severity in addition to the presence or absence of disease could be useful for physicians. Moreover, AI may be useful for preventing missed glaucoma diagnoses when glaucoma is complicated by other ophthalmic diseases; AI is also expected to help reduce medical costs. With all the possible benefits of this technology, AI is expected to be actively introduced into glaucoma management in the future. However, careful attention should be given to the potential impact of AI misjudgment on the practices of physicians, especially those who do not yet have high levels of experience.

Physician’s knowledge of and experience with AI are still insufficient. Although there were differences between the experts and trainees in this study in terms of feeling threatened by and their expectations of AI, the differences were not significant. Few participants from both the expert and trainee groups indicated that they were hardly affected by the AI judgment, while most reported being affected to some extent. Therefore, there is concern that the introduction of AI may cause confusion in clinical practice. One of the concerns of physicians about the introduction of AI in clinical practice is that the basis for AI decisions is unclear—the so-called black box problem [8,21]. Neural networks and deep learning-based AI algorithms lack clarity and transparency, which can make clinicians hesitant and uncertain when making prognostic and diagnostic decisions. Many physicians are reluctant to apply AI to clinical medicine, as the majority of current AIs only report judgment results without providing sufficient diagnostic evidence to convince physicians of these results. Hua et al. reviewed factors influencing the acceptability of AI in medical practice among physicians and emphasized that human-centered practice is essential [22]. These points all need to be considered before AI can be used in glaucoma practice.

Given that the number of evaluators in this study was relatively small, further studies should be conducted with a larger number of evaluators in the future. In the present study, the impact of the AI system was compared separately for experts and trainees, but it is possible that within each group, the diagnostic skills of the evaluators were not homogenous, which may have affected the results. In addition, the degree to which the evaluators deferred to the accuracy of the diagnosis of glaucoma by the AI system was not analyzed; therefore, the evaluation of the degree of influence may not have been sufficient. The fundus photographs were judged on the same computer in each trial by each evaluator, but the monitor’s resolution and other factors, including experimental circumstances, were not exactly the same among the evaluators; the possibility that these factors may have affected the judgment results cannot be completely ruled out. Although there was a sufficient interval between the first and second trials and the order of image presentation was changed randomly in the second trial, the possibility that a learning effect from the first trial affected the second trial cannot be completely eliminated. While visual field impairment is commonly used as a criterion for determining the severity of glaucoma, there is no clear consensus on its definition. In this study, the severity classification for training the AI system was based on the mean deviation (MD) value of the visual field, as described in the Methods section. Evaluators were instructed to adhere to this criterion when making their assessments.

## Conclusion

This study revealed that AI responses may influence ophthalmologists’ responses, especially among ophthalmologists with insufficient experience, but ophthalmologists need to fully understand that the AI system is not perfect. Improving physician knowledge about AI and providing a human-centered AI system for more adequate glaucoma management are important. Notably, the interaction between humans and AI in clinical settings should be balanced and subject to scrutiny as previously reported [23].
